# Synaptopodin: a key regulator of Hebbian plasticity

**DOI:** 10.3389/fncel.2024.1482844

**Published:** 2024-11-06

**Authors:** Pei You Wu, Yanis Inglebert, R. Anne McKinney

**Affiliations:** ^1^Department of Pharmacology & Therapeutics, McGill University, Montreal, QC, Canada; ^2^Department of Neurosciences, Université de Montréal, Montreal, QC, Canada; ^3^Centre Interdisciplinaire de Recherche sur le Cerveau et l’Apprentissage (CIRCA), Montreal, QC, Canada

**Keywords:** dendritic spines, synaptic plasticity, synaptopodin, mGluR-LTD, STDP, LTP, LTD

## Abstract

Synaptopodin, an actin-associated protein found in a subset of dendritic spines in telencephalic neurons, has been described to influence both functional and morphological plasticity under various plasticity paradigms. Synaptopodin is necessary and sufficient for the formation of the spine apparatus, stacks of smooth endoplasmic reticulum cisternae. The spine apparatus is a calcium store that locally regulates calcium dynamics in response to different patterns of activity and is also thought to be a site for local protein synthesis. Synaptopodin is present in ~30% of telencephalic large dendritic spines *in vivo* and *in vitro* highlighting the heterogeneous microanatomy and molecular architecture of dendritic spines, an important but not well understood aspect of neuroplasticity. In recent years, it has become increasingly clear that synaptopodin is a formidable regulator of multiple mechanisms essential for learning and memory. In fact, synaptopodin appears to be the decisive factor that determines whether plasticity can occur, acting as a key regulator for synaptic changes. In this review, we summarize the current understanding of synaptopodin’s role in various forms of Hebbian synaptic plasticity.

## Introduction

Dendritic spines are small thorn-like protrusions found on the dendrites of most of the excitatory neurons in the brain. They are the point of contact between neurons, forming the postsynaptic side of the excitatory synapses, and hold the molecular machinery that allows the transmission of signal from the afferent neurons (Gray, 1959; Hering and Sheng, 2001; Nimchinsky et al., 2002; McKinney, 2010). Spines can grow, shrink, form *de novo* and be maintained or be eliminated, all of which contribute to the formation and experience-dependent optimization of neuronal circuits (Caroni et al., 2012; Harris, 2020). These morphological modifications of spines are widely regarded as the structural basis for learning and encoding memories (Bourne and Harris, 2007; Kasai et al., 2021). They are also recognized as the loci of synaptic plasticity expression, believed to be the cellular mechanism underlying learning and memory formation (Hering and Sheng, 2001; Runge et al., 2020; McCann and Ross, 2017; Ma and Zuo, 2022). The most well-studied form of plasticity is Hebbian plasticity, in which the firing of one neuron induces the firing of another, strengthening the connection. Conversely, if the firing is desynchronized and does not drive the other neuron, the connection weakens. These processes correspond to the classical forms of long-term potentiation (LTP) and long-term depression (LTD), respectively.

The functional aspects of synaptic plasticity have been well characterized, whereby LTP increases synaptic strength and LTD decreases synaptic strength. Strengthened synaptic transmission during LTP is typically echoed by spine volume enlargement to accommodate more glutamate receptors (Bosch et al., 2014; Nakahata and Yasuda, 2018), as well as increased spine stability or spine density (Geinisman et al., 2001). Conversely, weakened synaptic transmission during LTD is typically associated with shrinkage and/or spine loss, synapse loss, and undesirable decreased connectivity in neuronal circuits (Nägerl et al., 2004; Zhou et al., 2004; Stein and Zito, 2019). Intriguingly not all dendritic spines will undergo similar plastic changes despite receiving a similar stimulus, highlighting a heterogeneity in synapses (Ramiro-Cortés and Israely, 2013; Szepesi et al., 2014; Thomazeau et al., 2021). In fact, spines are highly heterogeneous as they have been grouped into different subtypes based on their size and shape, which are often associated with their function in memory storage and formation (Papa et al., 1995; Hering and Sheng, 2001; Nimchinsky et al., 2002; von Bohlen und Halbach, 2009; McKinney, 2010; Rochefort and Konnerth, 2012; Pchitskaya and Bezprozvanny, 2020). Moreover, they also differ in terms of their molecular composition. Using electron microscopy (EM), Gray (1959) identified the presence of smooth endoplasmic reticulum (sER) in a subpopulation of the spines, and among those, some formed spine apparatus (SA), a more complex form of sER composed of several stacks of cisterns interconnected by electron dense materials (Frotscher et al., 2014). The formation and function of the sER in spines was a mystery, until recent live imaging experiments and 3D electron microscopy images revealed that the spine sER is continuous with the dendritic sER, which is critical for multiple forms of synaptic plasticity (Miyata et al., 2000; Holbro et al., 2009; Ostroff et al., 2010; Chirillo et al., 2019) and exhibits motility to enter or exit the spines (Špaček, 1985; Spacek and Harris, 1997; Perez-Alvarez et al., 2020).

The insertion of the sER in spines has been shown to be dependent on elevated synaptic transmission and activity and structural changes of spines after plasticity (Perez-Alvarez et al., 2020). The spines with stable sER have a longer lifetime with around 90% of these stable spines are associated with synaptopodin (SP), an actin-associated postsynaptic protein (Deller et al., 2000; Korkotian and Segal, 2011; Perez-Alvarez et al., 2020; Yap et al., 2020). While not expressed in the cerebellum, SP is found in the dendritic shaft, the dendritic spines and the axon initial segment of the excitatory neurons in the hippocampus, cerebral cortex and striatum (Mundel et al., 1997; Deller et al., 2000, 2003). In the hippocampus, SP expression is developmentally regulated as it gradually increases over the maturation of the brain circuitry, reaching its maximum in adult animals (Czarnecki et al., 2005). A similar developmentally regulated expression pattern was also observed in cultured neurons (Konietzny et al., 2019). SP is the only molecule found so far to be responsible for the formation of SA and colocalizes with it at the base of dendritic spines (Deller et al., 2003). SA is originated from sER, it is believed to act as an intracellular calcium reservoir and to help in the compartmentalization of calcium within spines (Buchs et al., 1994; Holbro et al., 2009; Korkotian and Segal, 2011; Korkotian et al., 2014; Rosado et al., 2022). Presence of ribosomes and translocon on SA also imply that it might play an essential role in local protein synthesis (Špaček, 1985; Pierce et al., 2000). Furthermore, the major glutamate receptors of the excitatory synapses, N-methyl-D-aspartate receptors (NMDAR) and *α*-amino-3-hydroxy-5-methyl-4-isoxazolepropionic acid receptor (AMPAR), were also found co-localizing with SA, suggesting a role in receptor turnover and trafficking (Nusser et al., 1998; Racca et al., 2000).

Studies on SP, the molecular marker of SA, have provided solid evidence that spines with or without SP are two functionally distinct groups of spines. SP is preferential localization in about 30% of the mushroom spine in adult hippocampus (Deller et al., 2003; Speranza et al., 2022b). SP expression in spines is also very dynamic and is differentially regulated by synaptic activity and various molecules such as the motor protein myosin V and miRNA (Yamazaki et al., 2001; Konietzny et al., 2019; Dubes et al., 2022). Presence or gain of SP in spines increases the spine head volume and the spine lifetime, whereas spines that lose SP have decreased spine size and survival time (Deller et al., 2000; Okubo-Suzuki et al., 2008; Zhang et al., 2013; Yap et al., 2020; Speranza et al., 2022b). Mice in which the *Synpo* gene encoding SP is deleted (SPKO) show deficits in spatial learning (Deller et al., 2003; Jedlicka et al., 2008), consistent with evidence that SP plays an important role in hippocampal structural/functional plasticity. SP regulates spine structural plasticity by supporting spine head enlargement induced by LTP (Okubo-Suzuki et al., 2008; Vlachos et al., 2009; Zhang et al., 2013; Chirillo et al., 2019). From our previous work we also know that under conditions of reduced activity, certain spines produce spine head protrusions to near by active presynaptic boutons, the stability of which depends on the presence of SP (Verbich et al., 2016). Increasing evidence in the past decade suggests that SP acts as a critical molecule for many forms of synaptic plasticity.

## NMDAR-LTP

**Figure 1:**
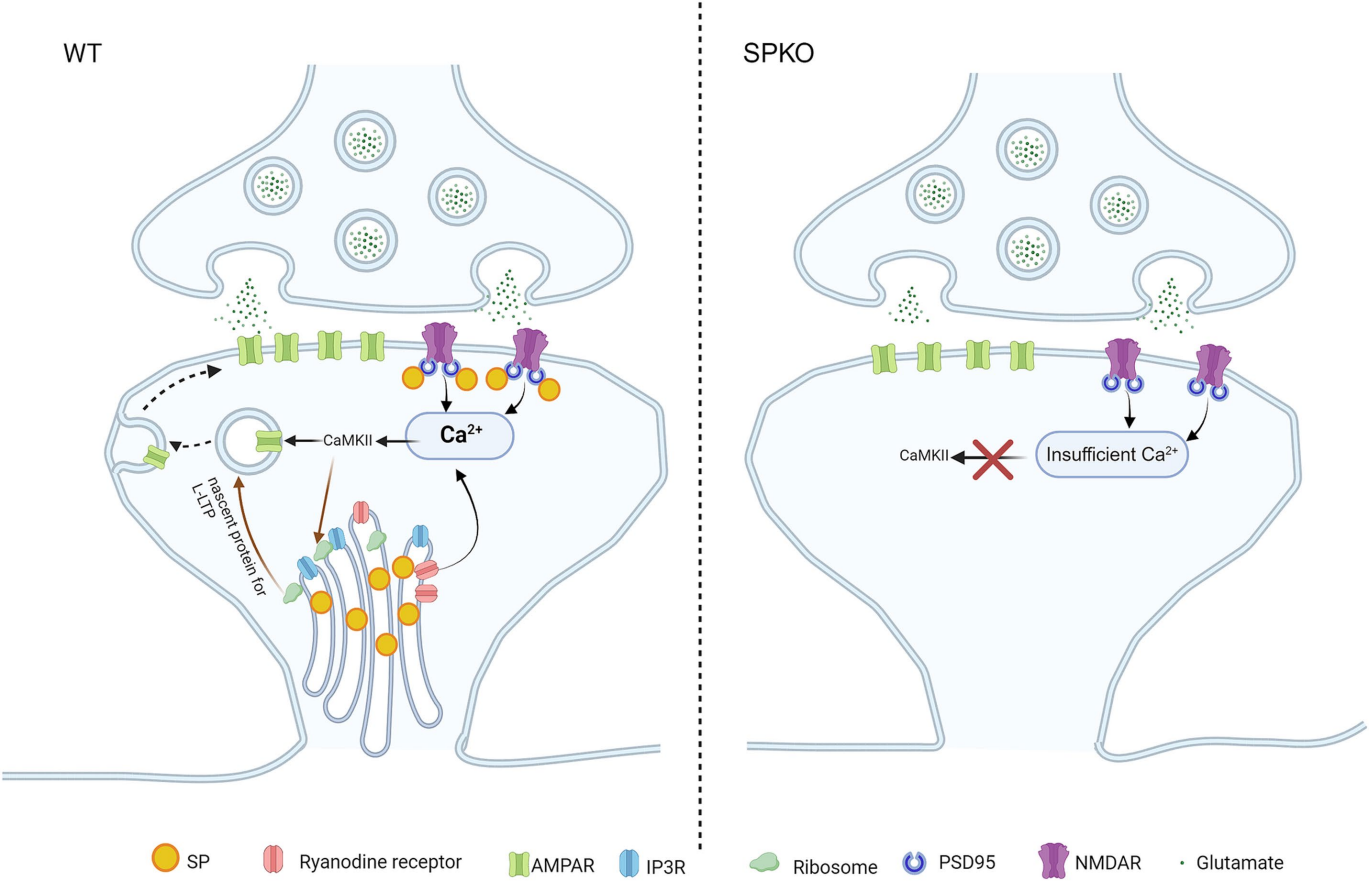
SP/SA is involved in Hebbian long-term potentiation. Presence of SP/SA in spines enables the release of Ca^2+^ from RYR and NMDAR, allowing for the build-up of high [Ca^2+^] in spines to activate CAMKII during NMDAR-LTP and t-LTP. Subsequent phosphorylation of AMPAR by CAMKII initiates AMPAR surface insertion (signaling pathway shown in black arrow). CAMKII can equally activate signaling pathways to induce the long-lasting form of LTP (l-LTP) through ribosome-mediated local *de novo* protein synthesis in WT, as ribosomes are associated with SA (signaling pathway shown in brown arrow). In SPKO, NMDAR-LTP and t-LTP are impaired as SA is absent in spines. Lack of SA causes insufficient release of Ca^2+^ in spines to activate CaMKII. Loss of SA also lead to removal of ribosome from spines, disabling local protein synthesis for l-LTP.

NMDAR -mediated LTP (NMDAR-LTP) is the most studied and best understood form of synaptic plasticity. It is commonly induced by either high frequency electrical stimulation (100 Hz) or chemical stimulation (e.g., NMDA, forskolin, etc.) (Dudek and Bear, 1992). The different stimulation methods may require slightly different molecular players, but NMDAR-LTP generally involves the elevation of post-synaptic calcium (Ca^2+^) through NMDAR, followed by the activation of CAMKII and Protein Kinase A (PKA), leading to the subsequent trafficking of *α*-amino-3-hydroxy-5-methyl-4-isoxazolepropionic acid receptor (AMPAR) to the post-synaptic density (PSD) membrane (Figure 1) (Chetkovich and Sweatt, 1993; Lüscher and Malenka, 2012). Insertion of AMPAR to the PSD increases the synaptic strength and is associated with dendritic spine enlargement and stability (De Roo et al., 2008; Holtmaat and Svoboda, 2009; Lüscher and Malenka, 2012; Kasai et al., 2021). Ultrastructural analysis of rat hippocampal CA1 dendritic spines following theta-burst stimulation induced LTP was shown to have enlarged PSD and perforated synapse, which is more likely to contain sER and spine apparatus (Toni et al., 2001; Chirillo et al., 2019). In fact, these post-LTP ultrastructural changes in dendritic spines were further confirmed after the discovery of SP, where studies showed that SP mRNA and protein expression as well as the dendritic spine size increase following LTP (Yamazaki et al., 2001; Fukazawa et al., 2003). The first observation of NMDAR-LTP deficits in SPKO was made in the hippocampus at Schaffer Collateral-CA1 (Sc-CA1) synapses, and was also linked to impairments in spatial learning (Deller et al., 2003). These findings have been subsequently confirmed by multiple independent studies (Zhang et al., 2013; Grigoryan and Segal, 2016; Inglebert et al., 2024), including the *in vivo* study in the Dentate Gyrus (Jedlicka et al., 2009). These studies provided solid evidence for a prominent role of SP in NMDAR-LTP, however how SP participates in the molecular mechanism of the plasticity is not well understood. An interesting observation has been made by Zhang et al., where only the younger SPKO mice (P15-21), but not the older mature mice (2 months- or 6 months-old), exhibits deficiency in NMDAR-LTP. The authors reasoned that the young developing mice require PKA activation for LTP and SP is a substrate of PKA (Yasuda et al., 2003; Faul et al., 2008; Zhang et al., 2013). In kidney podocytes phosphorylation of SP by PKA and CAMKII was found to protects from proteolysis and promotes stress fiber formation by stabilizing the GTPases RhoA (Asanuma et al., 2006; Faul et al., 2008), perhaps a similar mechanism occurs in the brain. However, direct evidence of SP phosphorylation and its relevant physiological effect in brain is still lacking. Despite this interesting observation by Zhang et al. (2013), a few studies showed conflicting results that NMDAR-LTP deficit is observed in mature SPKO mice (Table 1). Such discrepancy may be due to the difference in genetic background of the SPKO mice (Jedlicka and Deller, 2017), potential unknown compensatory mechanism in the adult animal, or the use of ventral hippocampal slices from WT, which exhibits weaker LTP than dorsal hippocampal slices, occluding the deficits in SPKO (Vlachos et al., 2008; Grigoryan and Segal, 2016). Lack of NMDAR-LTP in adult SPKO mice can be attributable to impaired CAMKII activation due to the loss of intracellular calcium store SA leading to insufficient [Ca^2+^] in spines.

**Table 1:** The importance of synaptopodin in synaptic plasticity.

Reference	Region	Synapse	Preparation	Protocol	Plasticity observed
Deller et al. (2003)	Hippocampus	Sc – CA1	Acute slices (adult)	TBS (100 Hz, 10 × 4 pulses) or tetani (3 × 30 pulses, 200 Hz)	Reduced LTP
Zhang et al. (2013)	Hippocampus	Sc – CA1	Acute slices (P15 or 21)	TBS (100 Hz, 10 × 4 pulses)	Reduced LTP
			Acute slice (2 or 6 month-old)	TBS (100 Hz, 10 × 4 pulses)	Normal LTP
			Acute slice (P15 or 21)	2 Hz, 10 min, 1,200 stimuli	Normal LTD
Grigoryan and Segal (2016)	Hippocampus	Sc – CA1	Acute slices (2 to 3 month-old)	100 Hz, 1 s	Reduced LTP
Inglebert et al. (2024)	Hippocampus	Sc – CA1	Acute slices (P15 to P21)	10 Hz, 900 stimuli	Reduced LTP
				100 Hz, 900 stimuli	Reduced LTP
				t-LTP (+10 ms, 0,3 Hz, 100 pairings)	Absence of LTP, LTD instead
				t-LTD (−25 ms, 0.3 Hz, 100 pairings)	Absence of LTD
				2 Hz, 900 stimuli	Absence of LTD
Jedlicka et al. (2009)	Hippocampus	PP – GC	Anesthetized mice (3 month-old)	TBS (4 × 15, 200 Hz) TBS (6 series of 6 × 6, 400 Hz)	Normal LTP
				TBS (6 series of 6 × 6, 400 Hz)	Reduced LTP
Speranza et al. (2022b)	Hippocampus	Sc – CA1	Acute slices (P30 – P40)	DHPG (100 μM, 5 min)	Reduced mGluR-LTD
Wu et al. (2024)	Hippocampus	Sc – CA1	Acute slices (P30 – P40)	S-DHPG (100 μM, 5 min)	Reduced mGluR-LTD
Vlachos et al. (2009)	Hippocampus	N/A	Hippocampal cultures	cLTP (Glycine)	Reduce GluR1 function
Korkotian et al. (2014)	Hippocampus	N/A	Hippocampal cultures	cLTP (Glycine)	Absence of morphological plasticity

This table summarizes the plasticity observed in the absence of synaptopodin under various protocols. It details the region, synapses, and type of preparation used. Additionally, the frequency of stimulation and the number of repetitions are specified. For instance, “10 × 4 pulses” means that the 4 pulses are repeated 10 times. Abbreviations used include TBS for Theta Burst Stimulation, t-LTP for timing LTP, Sc for Schaffer Collateral, PP for Perforant Path, GC for granule cells and cLTP for Chemical LTP.

In addition to electrophysiological data, SP has been shown to regulate the structural plasticity of dendritic spines. Specifically, SP is necessary for NMDA-induced spine expansion (Zhang et al., 2013) and broadly regulates dendritic spine plasticity (Vlachos et al., 2009). During NMDAR-LTP in WT mice, SP is upregulated in neurons and is specifically recruited to dendritic spines, where it promotes the enlargement of the spines and the accumulation of the AMPAR subunit GLUA1 in the spines (Yamazaki et al., 2001; Vlachos et al., 2009; Korkotian et al., 2014). It is possible that SP also regulates actin dynamics through the Rho GTPases in neurons, in a way similar to how SP regulates stress fiber formation in kidney podocytes, to mediate the structural plasticity of the spines (Asanuma et al., 2006). There is currently a lack of information of the involvement of SP in actin dynamics in the brain and will be an important area for future research.

**Figure 2:**
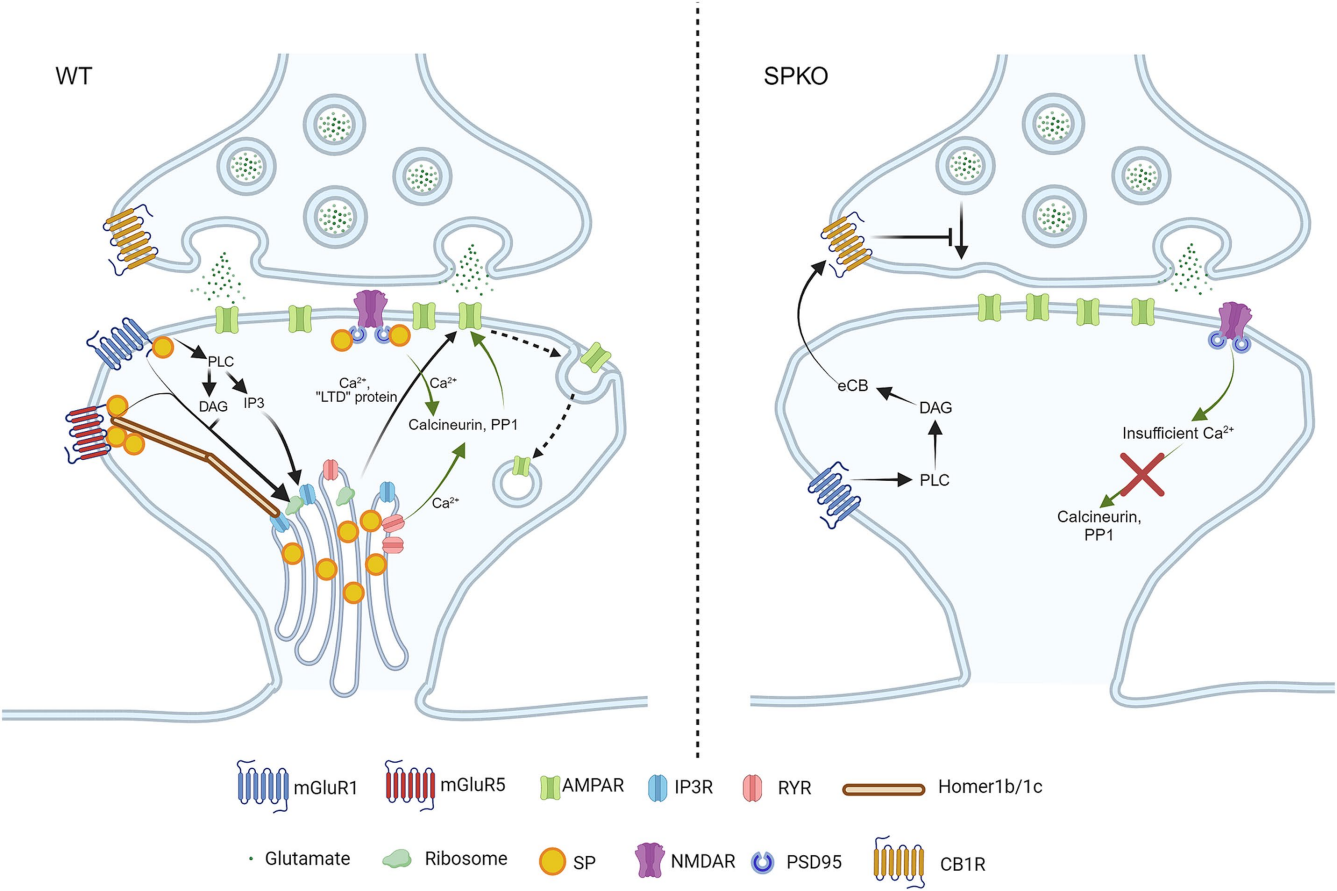
SP/SA is necessary for Hebbian long-term depressions. In WT spines that contain SP/SA, mGluR5 is stabilized by long Homers in the presence of SP. Upon activation of mGluR1/5, they trigger the release of phospholipase C (PLC), diacylglycerol (DAG) and Inositol trisphosphate (IP3) to activate IP3R and ribosomes. IP3R and ribosomes are found on SA, and they release Ca^2+^ and produce “LTD proteins” in order to promote endocytosis of AMPAR (signaling pathway shown in black arrows). NMDAR-LTD requires calcium entry from both NMDAR and RYR to activate the calcium-sensitive enzymes (calcineurin, PP1), to induce AMPAR internalization (signaling pathway shown in green arrows). In SPKO, due to the absence of SP and SA in spines, the postsynaptic mechanism of mGluR-LTD is completely switched to presynaptic mechanism through endocannabinoid signaling. The endocannabinoid is produced by mGluR1 alone, due to the loss of mGluR5 in the absence of SP, and retrograde signals back to presynaptic CB1R, leading to decreased glutamate release. As for NMDAR-LTD, the loss of RYR in the absence of SP/SA impairs intracellular Ca^2+^ release, leading to improper enzymatic signaling.

The loss of SP also affects the long-lasting maintenance of LTP (lasting from hours to months) (l-LTP) (Zhang et al., 2013), which relies on local protein synthesis (Otani et al., 1989; Raymond et al., 2000; Kelleher et al., 2004). Electron micrographs have revealed the presence of polyribosomes and translocon in close association with SA, and that spines containing SP/SA are more likely to contain ribosomes compared to spines devoid of SP/SA, suggesting a critical role of SP in regulating local protein synthesis (Špaček, 1985; Pierce et al., 2000; Kruse et al., 2024). Complete loss of SP (e.g., SPKO) may significantly affect ribosomal trafficking and localization in spines, leading to the impairment of plasticity mechanisms that require *de novo* local protein synthesis, including l-LTP and mGluR-LTD, which will be elaborated later in this review (Figures 1, 2). Recent discovery of monosomes in synapses contributing to the local synthesis of many key synaptic proteins adds an additional layer of complexity in understanding the regulation of local protein synthesis in spines (Biever et al., 2020). It would be interesting to uncover the relationship between SP and monosomes and perform an in-depth study on SP/SA’s role in synapse-targeting and regulation of protein synthesis machinery in dendritic spines, the primary sites of protein production (Figure 1).

## NMDAR-LTD

In contrast to LTP, the role of SP in LTD has been less explored. One primary reason is that low-frequency stimulation at 2 Hz yields conflicting results. At Sc-CA1 synapses, earlier findings indicated that LTD is normal in SPKO mice (Zhang et al., 2013), while our recent data showed that LTD is absent (Inglebert et al., 2024). This discrepancy can be attributed to variations in the number of stimulations, the extracellular Ca^2+^ concentration or the genetic background of the animals, similar to the discrepancy observed in adult LTP studies (Zhang et al., 2013; Grigoryan and Segal, 2016). It is important to note that LFS-LTD can be induced at various frequencies (usually between 1 and 10 Hz), but so far has only been explored at 2 Hz in SPKO mice. The lack of LTD in SPKO observed in Inglebert et al. (2024) is supported by the fact that ryanodine receptors (RYR) level is significantly reduced in spines of SPKO, as RYR-mediated calcium has been shown to play an important role in LTD (Arias-Cavieres et al., 2018). This could lead to insufficient Ca^2+^ entry into spines to activate calcineurin and protein phosphatase 1, resulting in disrupted signaling and impaired AMPAR endocytosis (Figure 2). This recent evidence calls for further investigation into the role of SP in activity-dependent LTD, either induced by LFS or STDP.

## Metabotropic glutamate receptors-LTD (mGluR-LTD)

**Figure 3:**
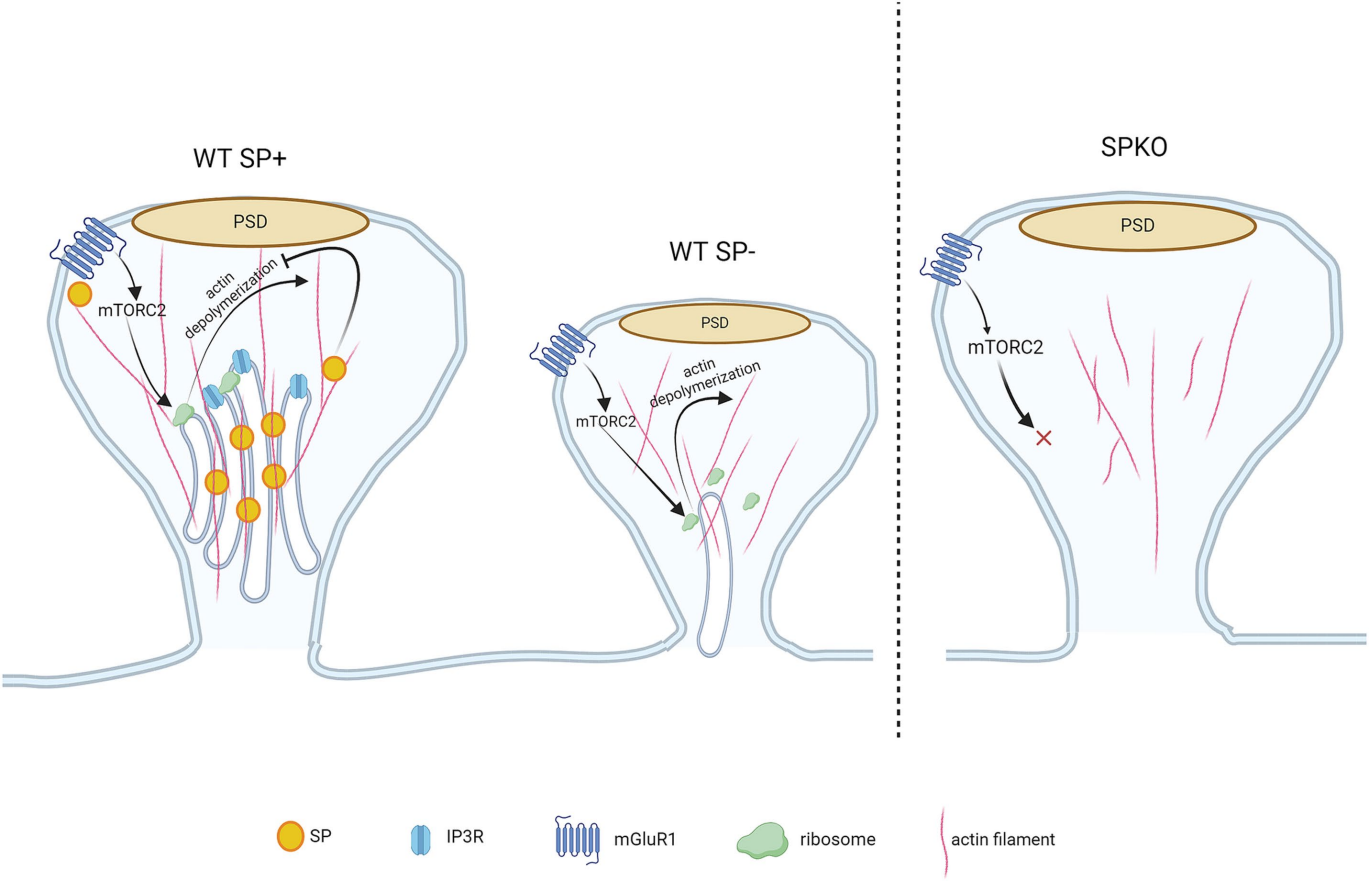
mGluR-LTD-mediated spine structural remodeling is regulated by synaptopodin. WT spines containing SP are protected from mGluR-LTD-induced shrinkage due to the presence of SP/SA that binds to F-actin and prevents actin depolymerization. WT spines lacking SP undergo spine shrinkage as SP is absent in the spine, allowing the F-actin to be depolymerized following mGluR1-induced mTORC2 signaling. Though the spine is devoid of SP/SA, they contain sER and protein synthesis machinery required for structural remodeling (i.e., mTORC2 and ribosomes). The SPKO spines do not display mGluR-LTD-dependent structural plasticity. They lack SP/SA, sER and the molecular machinery that is required for mGluR-LTD-induced structural plasticity.

One specific type of LTD, known as mGluR-LTD, is mediated by group 1 metabotropic glutamate receptors (mGluR1 and mGluR5). This form of LTD is observed in various brain regions, both *in vitro* and *in vivo*, including the hippocampus, amygdala, cortex, striatum and cerebellum (Bolshakov and Siegelbaum, 1994; Oliet et al., 1997; Palmer et al., 1997; Lüscher and Huber, 2010). It is typically induced by a brief application (5–10 min) of the mGluR agonist R,S-Dihydroxyphenylglycine (DHPG), which can lead to either the removal of postsynaptic AMPARs or a decrease in presynaptic release, depending on the concentration of DHPG (Sanderson et al., 2022). In mature neurons, AMPAR endocytosis by the rapid local translation of “LTD proteins” such as Arc, Map1b and STEP is a hallmark of mGluR-LTD (Figure 3) (Waung and Huber, 2009). In addition, mGluR-LTD induces structural changes which are characterized by spine shrinkage or loss (Oh et al., 2013; Ramiro-Cortés and Israely, 2013). Yet, spines loss is not always observed (Thomazeau et al., 2021). Since the ability of Gp1 mGluRs to depress synaptic transmission depends on sER (Holbro et al., 2009), It has been hypothesized that the presence of SP might explain the discrepancies observed in the literature. Indeed, in hippocampal slices from SPKO, both functional and structural mGluR-LTD is impaired (Speranza et al., 2022b; Wu et al., 2024). Notably, the application of DHPG results in the selective loss of mushroom-shaped spines that lack SP through mGluR1 but not mGluR5 activity, while spines containing SP remain stable (Speranza et al., 2022b).

Spine shrinkage/loss may occur through the mammalian target of rapamycin complex 2 (mTORC2)-signaled protein synthesis, which has been shown to regulate mGluR-LTD, actin re-organization and protein synthesis (Huang et al., 2013; Takei and Nawa, 2014; Huber et al., 2015; Zhu et al., 2018). Spines containing SP are protected from structure remodeling during mGluR-LTD due to the presence of SP in spines that physically binds and tethers the actin filaments (Figure 3). Interestingly, SPKO mice do not exhibit significant spine elimination despite unchanged mGluR1 expression. This suggests that the activation of downstream effectors, such as protein synthesis machinery, may be defective or absent in SPKO mice (Špaček, 1985; Pierce et al., 2000; Wu et al., 2024). In contrast to structural plasticity, the decrease in synaptic strength requires the combined activity of both mGluR1 and mGluR5 (Volk et al., 2006; Wu et al., 2024). Although mGluR1 surface expression is normal in SPKO mice, mGluR5 surface expression is reduced, explaining the observed impairment in mGluR-LTD. Loss of SP destabilizes the interaction between scaffolding proteins (such as long Homers) that keeps mGluR5 anchored on the surface of dendritic spines (Brakeman et al., 1997; Wu et al., 2024) (Figure 2). More surprisingly, we found that the residual mGluR-LTD in SPKO mice is protein synthesis-independent and is instead mediated by a decrease in presynaptic release through endocannabinoid signaling as observed in neonatal animals (P8-15) (Nosyreva and Huber, 2005; Wu et al., 2024). This suggests that SP may regulate the locus of expression of mGluR-LTD and that loss of SP probably impact the normal maturation of the hippocampus.

## Spike timing-dependent plasticity

Spike Timing-Dependent Plasticity (STDP) is an activity-dependent plasticity thought to be more physiological and closer to what could happen in the brain (Debanne and Inglebert, 2023). Unlike plasticity dependent on stimulation frequency, STDP relies on the precise timing between presynaptic activity (excitatory postsynaptic potential, EPSP) and postsynaptic activity (action potential, AP). Classically, timing-dependent LTP (t-LTP) is induced when an EPSP is followed by an AP in the postsynaptic neuron and timing-dependent LTD (t-LTD) is induced when AP is followed by an EPSP (Bi and Poo, 1998; Debanne et al., 1998). SP has been recently identified to be required for normal t-LTP and t-LTD at Sc-CA1 synapses (Inglebert et al., 2024). The absence of SP shifts t-LTP into t-LTD but can still be restored by adjusting the parameters of the STDP paradigm (Table 1). The higher threshold for t-LTP in SPKO mice is most likely due to the loss of AMPAR in Sc CA1 synapses, which works in conjunction with NMDAR to induce postsynaptic Ca^2+^ elevation in spines. Loss of AMPAR might have caused a reduction of Ca^2+^ in spines resulting in impaired t-LTP. A stronger STDP protocol that induced stronger NMDAR-mediated currents might have overcame the impact of AMPAR deficiency and therefore rescued the t-LTP deficit (Inglebert et al., 2024; Nevian and Sakmann, 2006) (Figure 1). In the hippocampus, at Sc-CA1 synapses, t-LTD has been described to required numerous molecular players to exist, such as calcium release from internal stores and mGluR5 activation, which are both disrupted in the absence of SP (Inglebert et al., 2024; Wu et al., 2024) (Figure 2). Since STDP can be induced at the single spine level by pairing glutamate uncaging with post-synaptic APs (Tazerart et al., 2020), it presents a compelling paradigm for studying the role of SP in morphological plasticity of dendritic spines. Remarkably, following glutamate uncaging on SP-positive spines, neighboring synapses demonstrate spine head shrinkage (Korkotian et al., 2014). *Could SP-positive spines influence neighboring spines more strongly or differently than SP-negative spines*? In support of this idea, after TBS-induced LTP, the vicinity of spines expressing sER formed larger clusters with an increased total synaptic weight. We believe that STDP provides an ideal framework for investigating both homo- and hetero-synaptic plasticity at the single-spine level, as it is highly dependent on clustered dendritic spines. For example, t-LTP can be enhanced through the co-activation of closely clustered spines (within <5 μm) (Tazerart et al., 2020). The presence of SP in the spine could alter the rules of synaptic cooperation and is an interesting area for future studies. In addition, in some cases, STDP also necessitates activation of group 1 metabotropic glutamate receptors (Group I mGluRs), which we recently demonstrated to be down-regulated in SPKO mice (Speranza et al., 2022b; Wu et al., 2024).

## Synaptopodin and calcium dynamics

SP/SA has long been identified as an important source of postsynaptic Ca^2+^ and regulates Ca^2+^ dynamics within spines. In fact, experiments involving glutamate uncaging at single spines have demonstrated that, regardless of spine shape, SP-positive spines exhibit stronger calcium transients compared to SP-negative spines (Korkotian and Segal, 2011; Korkotian et al., 2014). This elevated calcium signal likely originates from intracellular Ca^2+^ stored in the SA/sER, as the application of thapsigargin or cyclopiazonic, the sER calcium depletion agent, eliminates this difference. Specifically, RYR are considered key players in the release of Ca^2+^ from internal stores. Indeed, SP expression is correlated with the presence of RYR, and the specific application of caffeine, a RYR agonist, results in a greater Ca^2+^ increase in SP-positive spines (Vlachos et al., 2009; Segal et al., 2010). This Ca^2+^-induced Ca^2+^-release from internal stores has been shown important for structural plasticity as blocking the RYR or depleting the intracellular calcium store was shown to prevent the structural expansion and AMPAR accumulation in spines (Vlachos et al., 2009). In addition, blocking of RYR have been long shown to prevent the expression of different forms of LTP as well as NMDAR-LTD at Sc-CA1 synapses (Raymond and Redman, 2002; Mellentin et al., 2007; Grigoryan et al., 2012; Arias-Cavieres et al., 2018; Valdés-Undurraga et al., 2023). Similarly, t-LTP has been shown to require RYR signaling depending on the repeat number and frequency of the STDP stimulation (Cepeda-Prado et al., 2022). Furthermore, knocking down SP significantly reduces RYR-positive spines and prevents the accumulation of GLUA1 in spines (Vlachos et al., 2009). RYR are not the only molecular players expressed in the SA/sER that control calcium dynamics and affect plasticity. Inositol triphosphate receptor (IP3R) mediated Ca^2+^ transient was observed only in the SA/sER-containing spines, and is necessary for NMDAR-LTP/LTD as well as mGluR-LTD (Taufiq et al., 2005; Holbro et al., 2009; Yoshioka et al., 2010). In addition, store-operated calcium entry (SOCE) channels, Orai1/STIM1, which serve to replenish the Ca^2+^ stores when depleted, are also preferentially located in SP-positive spines and may contribute to the amplified calcium response in these SP-positive spines (Korkotian et al., 2014). Based on these experimental evidence, SP/SA plays a central role in regulating postsynaptic Ca^2+^ dynamics during activity. SP/SA-positive spines, where intracellular calcium signaling is present, appear to be the locus of expression for synaptic plasticity. The deficits in these different forms of Hebbian plasticity in SPKO that have been discussed earlier in this review can all be partially, if not completely, attributable to the impaired calcium signaling. Additionally, recent publications provided compelling evidence showing that postsynaptic calcium dynamics regulates local translation in a synaptic plasticity-specific manner, implying calcium as the central signaling molecule involved in the induction and maintenance of synaptic plasticity (Sun et al., 2021; Ramakrishna et al., 2024). This suggests that the protein synthesis pathways, which are implicated in many forms of Hebbian plasticity and often considered as an independent mechanism to the calcium signaling, are downstream effectors regulated by calcium dynamics. Based on this idea, SP/SA, the master switch of calcium in dendritic spines, would be the ultimate regulator of Hebbian plasticity. Moving forward, experiments combining calcium imaging and electrophysiology as well as biochemical analysis of calcium-dependent proteins are necessary to gain a better understanding of calcium dynamics and calcium signaling in SP-positive spines during the activity-dependent plasticity.

## Synaptopodin and neuronal excitability

Synaptopodin is also expressed in the Axon Initial Segment (AIS), where action potentials are generated (Schlüter et al., 2019). Plasticity in the AIS serves as a powerful regulatory mechanism for neuronal excitability, as changes in morphology (*such as length and distance from the soma*) or ion channel expression can lead to increased or decreased excitability (Yamada and Kuba, 2016), directly affecting synaptic plasticity induction threshold. CA1 pyramidal neurons from 6-month-old SPKO mice show increased intrinsic excitability (measured by field potential recordings) and altered spike waveform properties (Aloni et al., 2021). However, the mechanisms underlying this increased excitability remain largely unknown. It is speculated that it may serve as a homeostatic compensatory mechanism to counteract reduced synaptic plasticity. Additionally, it is important to note that no study has thoroughly characterized the intrinsic excitability in SPKO mice, including parameters such as input–output curves or rheobase. Consequently, the impact of SP loss on intrinsic excitability remains uncertain. One possible explanation is that it directly influences the development of the AIS, as suggested by several studies. For instance, following LTP in hippocampal granule cells, AIS shortening is linked to a reduction in SP clusters (Jungenitz et al., 2023) and the absence of SP impairs the maturation of AIS length in the visual cortex (Schlüter et al., 2017). Furthermore, recent evidence suggests that the AIS can rapidly shorten following LTD in the hippocampus (Fréal et al., 2023), though the role of SP in this process remains unclear.

## Synaptopodin in neurological disorders

While SP is heavily implicated in the cellular form of learning by mediating various forms of synaptic plasticity in brain, not much is known about SP role in the diseased state of the brain. Many neurodevelopmental and neurodegenerative disorders have been shown deficits in synaptic activities and plasticity (Usdin et al., 1999; Huber et al., 2002; Ma et al., 2010; Yoo et al., 2014; Chu, 2020). Recently, SP has been identified to be linked to autism spectrum disorder and regulates calcium dynamics as well as spine structural plasticity in a mouse model of autism (Hu et al., 2023). Furthermore, in a mouse model of Fragile X syndrome (FXS), which is the most prevalent form of intellectual disability and has enhanced mGluR-LTD, SP was found upregulated in the dendritic spines, especially in the long thin type of immature spines that usually do not contain SP (Huber et al., 2002; Speranza et al., 2022a), As the presence of SP in spines enhance spine stability, the upregulation of SP in spines of FXS mouse model could explain the increase of in total and thin spine density in these mice (Comery et al., 1997; McKinney et al., 2005; Speranza et al., 2022a). Together with the deficit of mGluR-LTD in SPKO (Wu et al., 2024), this finding suggests that SP level requires precise regulation, too much or too little SP could both lead to abnormal synaptic plasticity and potentially neurological disorders. SP has been shown to be required for lesion-induced synaptic homeostatic changes following neuronal denervation, which is often resulted from demyelination, cells death and traumatic brain injury (Vlachos et al., 2013; Kruse et al., 2024). SP’s ability to modulate calcium has been used to rescue Alzheimer’s disease in mouse model. Aloni et al. (2019) has crossed SPKO mice with the 3xTg Alzheimer’s mouse model in the attempt of rescuing the LTP deficit that was observed in the Alzheimer’s mouse model. This was achieved by decreasing the intracellular calcium level that is elevated in the disease mouse model (Chakroborty et al., 2009). Not only did the LTP deficit was rescued, animal spatial learning was also improved in the crossed mice. Current data on SP involvement in diseases is only just beginning and is very limited, however current data suggests that SP has a significant clinical implication. Future studies on SP will reveal more exciting findings on the role of SP on memory formation and storage as well as its role in neurological diseases.

## Concluding remark

This review highlighted the recent findings and discovery about SP’s role in Hebbian synaptic plasticity. SP/SA, acting as the important source of Ca^2+^ in the postsynaptic compartment, regulates virtually all types of Hebbian plasticity. Increasing studies start to look at SP from different perspectives. Proteomic studies have identified novel binding partners of SP in spines, further delineating SP/SA function in spines (Falahati et al., 2022; Konietzny et al., 2023). Electrophysiological studies showed SP playing important roles in non-Hebbian plasticity as well (Vlachos et al., 2013; Dubes et al., 2022). A key question that has never been answered for SP is why it is only present in a subset of dendritic spines and what determines its presence in spines. Some may argue activity determines its presence in spines, but SP is also regulating the activity of the spines. Future studies are needed to identify the missing factor that determines the heterogeneous presence of SP in spines.
